# Rho-GEF Trio regulates osteosarcoma progression and osteogenic differentiation through Rac1 and RhoA

**DOI:** 10.1038/s41419-021-04448-3

**Published:** 2021-12-11

**Authors:** Junyi Wang, Lichan Yuan, Xiaohong Xu, Zhongyin Zhang, Yuhuan Ma, Leilei Hong, Junqing Ma

**Affiliations:** 1 Jiangsu Key Laboratory of Oral Diseases, Nanjing Medical University, 140 Hanzhong Road, 210029 Nanjing, China; 2 Nanjing Foreign Language School, 210008 Nanjing, Jiangsu China

**Keywords:** Bone cancer, Bone cancer

## Abstract

Osteosarcoma (OS) is the most common primary bone tumor. Its high mortality rate and metastasis rate seriously threaten human health. Currently, the treatment has reached a plateau, hence we urgently need to explore new therapeutic directions. In this paper, we found that Trio was highly expressed in osteosarcoma than normal tissues and promoted the proliferation, migration, and invasion of osteosarcoma cells. Furthermore, Trio inhibited osteosarcoma cells’ osteogenic differentiation in vitro and accelerated the growth of osteosarcoma in vivo. Given Trio contains two GEF domains, which have been reported as the regulators of RhoGTPases, we further discovered that Trio could regulate osteosarcoma progression and osteogenic differentiation through activating RhoGTPases. In summary, all our preliminary results showed that Trio could be a potential target and prognostic marker of osteosarcoma.

## Introduction

Osteosarcoma (OS), as one of the common malignant bone tumors, is derived from osteoblasts and mainly affects adolescence and childhood [1, 2]. It mostly occurs in the long bones, additionally 6–10% in the craniofacial bones, causing jawbones lesions and facial deformities [1, 3]. With the aid of neoadjuvant and adjuvant chemotherapy, the 5-year survival rate of localized OS has increased to 70% since the 1970s [4, 5]. Currently, new molecular imaging methods, improved surgical techniques, and improved implant design provide convenience for precision surgery [6–8]. Of note, the 5-year survival rate deteriorates to 20% for metastatic patients [4, 5]. Some studies put forward that the bone microenvironment, such as mesenchymal stem cells, hypoxia, and chemokines, may be used as the potential target for lung metastasis [9, 10]. Besides, immunotherapy has proven to be a promising therapeutic strategy which intended to block molecular pathways associated with proliferation and metastasis of OS [7, 11]. Even with multiple advanced treatment therapies, effective therapeutic targets or diagnostic markers for OS have not yet been determined. No significant progress has been made in the treatment of OS, which makes its treatment more intractable and challenging. Therefore, further research is needed for progressive strategies and novel treatment targets of OS.

RhoGTPases belong to the Ras superfamily of GTPases, among which RhoA, Rac1, and Cdc42 are the three most canonical members [12]. RhoGTPases are implicated in a series of cellular events, including cell polarity, adhesion, motility, and cycle progression by regulating actin cytoskeleton rearrangements and microtubule dynamics. Consequently, Rho GTPases have active roles in malignant transformation, immunological diseases, neurological abnormalities, and cardiovascular diseases [12, 13]. It has been established extensively that RhoGTPases switch back and forth between an active GTP-bound state and an inactive GDP-bound state. There are three canonical regulators that can affect this switching regulation of GDP/GTP, including Rho family guanylate exchange factors (GEFs) that accelerate the conversion of GDP to GTP, rendering the GTPases active; GTPases activating proteins (GAPs) that promote the hydrolysis of GTP and inactivate GTPases; GDP dissociation inhibitors (GDIs) that sequester the inactive GDP-bound GTPases in the cytoplasm and inhibit them from being activated by GEFs [12–14].

Triple functional domain (Trio), which is the member of the DBL family, harbors three domains with enzymatic activity, two GEFs, and one serine kinase domain. The GEFD1 domain can activate Rac1 as well as RhoG, while the GEFD2 domain specifically activates RhoA. In addition, the activated RhoG can further activate Cdc42 [15]. The Trio is well-known for its central player in neuronal development [15–17]. Moreover, Trio also plays a prominent role in fetal skeletal muscle formation and embryonic development [18]. In recent years, a mounting number of studies have demonstrated that Trio is significantly increased in different types of tumors, including bladder cancer, breast cancer, lung cancer, oral cancer, glioblastoma, and soft tissue sarcoma [15, 19–21]. However, the regulatory mechanism responsible for the development of tumors has not yet been fully elucidated, and little is known either about the roles of Trio in OS. Currently, a number of studies have confirmed that RhoGTPases or their downstream molecules are instrumental in the invasion and metastasis of OS cells [14, 22–29]. Accordingly, we put forward the hypothesis that Trio could regulate OS cells migration and invasion through RhoGTPases.

We previously showed that Trio had important functions both in regulating tooth root development and osteoclast differentiation [30, 31]. In this study, we explored the influences of Trio on OS cells and the potential biochemical mechanism regulated by Trio. In my view, this study is beneficial in providing new directions for the treatment and prognosis of OS.

## Materials and methods

### Cell culture

Normal human osteoblast cell line hFOB1.19, as well as human OS cell line U2OS, were purchased from GeneChem (Shanghai, China). Human OS cell line 143B was obtained from FuHeng Cell Center (Shanghai, China). U2OS and 143B were cultured in Dulbecco’s modified Eagle’s medium (DMEM) supplemented with 10% FBS. hFOB1.19 was cultured in F12/ DMEM supplemented with 15% FBS. All of the above cell lines were cultured in an incubator containing 5% CO_2_ at 37 °C.

### Clinical samples

OS tissue samples and their matched normal samples from 10 OS patients were collected from Jiangsu Provincial Stomatological Hospital and informed consent was obtained from all patients. The Ethical Committee at the Stomatological School in Nanjing Medical University approved this study.

### Cell migration and invasion assays

To assess the migratory and invasive ability of OS cells, we conducted the transwell assay with a chamber (8 µm; Corning; for the migration assay) or a chamber precoated with Matrigel (Corning, 356234; for the invasion assay). Briefly, U2OS and 143B cells (5 × 10^4^ for the migration assay or 10^6^ for the invasion assay) were seeded on the upper chambers of 24-well plates. Then the 500 μL medium supplemented with 10% FBS was added in the lower chamber as a chemoattractant. After 24 h incubation, the cells that had migrated or invaded into the lower chambers were fixed with 4% paraformaldehyde (PFA) for 30 m, then stained with crystal violet for another 15 m and finally observed by microscopy (Leica Microsystems, Ontario, Canada) with ×200 magnification. Three fields were randomly selected to count cell numbers.

### Wound healing assay

U2OS and 143B cells were seeded at a density of 5 × 10^5^ cells/ well into six-well plates and cultured until a 90–100% confluence was formed. We then scratched the cells using 200 µL pipette tips. Floating cells were washed once with PBS, and photos were taken by a light microscope (DMIL LED; Leica Microsystems GmbH) at 0, 24, and 48 h with ×100 magnification.

### CCK-8 assay

Cell-counting kit-8 (Dojindo, Japan) was conducted in order to evaluate the cell proliferation ability. Cells (10^3^) were seeded in 96-well plates and the medium was replaced with 90 μL fresh medium added with 10 μL CCK-8 reagent at the scheduled time points (0, 24, 48, 72, and 96 h). Following 2 h incubation, the absorbance was measured by the Microplate reader at an optical density of 450 nm.

### Colony formation assay

U2OS and 143B cells (10^3^) were seeded in six-well plates and cultured for 10 days to form colonies. Then, the cells were washed twice with PBS, fixed with 4% PFA, and stained with crystal violet for 15 m.

### Flow cytometry

To measure apoptosis, cells were trypsinized, washed with PBS, filtered with filter membranes, and stained with Annexin V-FITC/PI. Cell apoptosis was analyzed by a flow cytometer (Becton-Dickinson, SanJose, CA, USA).

### Small interfering RNA (siRNA) transfection

Three kinds of siRNAs that targeted Trio and negative control siRNA were purchased from GenePharma (Suzhou, China). The siRNAs sequences are included in Table 1.

**Table 1:** List of siRNAs sequences.

	Forward (5′–3′)	Reverse (5′–3′)
1#	CCGGCAAACUUGGAUCCAUTT	AUGGAUCCAAGUUUGCCGGTT
2#	GCAGACGUCUUCCUGAAAUTT	AUUUCAGGAAGACGUCUGCTT
3#	CCUUCAACCCUUCGGAUAATT	UUAUCCGAAGGGUUGAAGGTT

Lipofectamine 2000 (Invitrogen, CA) was used to transfect U2OS and 143B cells with siRNAs according to the manufacturer’s instructions when grew to a 50–70% confluence. After transfection for 48 h, cells were collected and prepared for further assays.

### Lentiviral transfection

A lentivirus targeting Trio and a negative control lentivirus were purchased from GenePharma (Suzhou, China). The shRNA Trio sequences were as follows:

shTrio-1: 5′-GCAGACGTCTTCCTGAAAT-3′;

shTrio-2: 5′-CCTTCAACCCTTCGGATAA-3′.

After 72 h infection with lentivirus, stably transfected cells were selected using puromycin (2 μg/ml) for 2 weeks. The infection efficiency was observed in a form of GFP by fluorescence microscopy (Leica Microsystems, Ontario, Canada) and verified by western blot.

### IHC and H&E staining assay

The paraffin-embedded samples were sectioned (3 μm thickness) for further immunohistochemistry and H&E assays. For immunohistochemistry, the tissue sections were firstly dewaxed and boiled in sodium citrate buffer solution for 10 min to retrieve antigens, subsequently treated with 3% H_2_O_2_ for 30 m and blocked with normal goat serum for 30 m at 37 °C. Finally, sections were incubated with the primary antibody at 4 °C overnight. The next day, sections were washed with PBS, then treated with a secondary HRP-conjugated antibody and dyed using the DAB developing kit. Eventually, cell nuclei were stained with hematoxylin. For H&E, the dewaxed sections were stained with hematoxylin for cell nuclei and eosin for cytoplasm.

The following antibodies were used: Trio (Santa Cruz, sc-28564), E-Cadherin (Cell Signaling Technology, #3195), N-Cadherin (Cell Signaling Technology, #13116), Vimentin (Cell Signaling Technology, #5741), RUNX2 (Abcam, #ab76956), OSX (Abcam, #ab22552), OPN (Abcam, #ab63856).

### Western blotting analysis

Cell proteins were extracted as previously described [32]. Equal levels of proteins were loaded onto 10% SDS–PAGE gel for electrophoresis (6% for Trio). After the electrophoresis, proteins were transferred to PVDF membranes on ice and immediately blocked with 5% defatted milk (5% BSA for phosphorylated protein) for 2 h at room temperature, finally incubated with primary antibodies at 4 °C overnight. On the second day, the membranes were washed in TBST three times, then incubated with secondary antibodies for 1 h at room temperature and washed with TBST another three times. Eventually, the blots were detected by an ECL chemiluminescence system. Quantitative analysis of images was conducted using ImageJ v.1.52 software.

The following antibodies were used: Trio (Abcam, #ab194365), E-Cadherin (Cell Signaling Technology, #3195), N-Cadherin (Cell Signaling Technology, #13116), Vimentin (Cell Signaling Technology, #5741), Snail (Cell Signaling Technology, #3879), RUNX2 (Abcam, #ab76956), OSX (Abcam, #ab22552), OPN (Abcam, #ab63856), OCN (Abcam, #ab93876), p38 (Cell Signaling Technology, #9212), p-p38 (Cell Signaling Technology, #9211), JNK (Cell Signaling Technology, #9252), p-JNK (Cell Signaling Technology, #4668,), ERK (Cell Signaling Technology, #4695), p-ERK (Cell Signaling Technology, #4370), GAPDH (Bioworld, #AP0063).

### RNA isolation and quantitative real-time PCR

Total RNA was extracted by an RNA isolation kit (BioTeke, Beijing, China) and cDNAs were synthesized by HiScript^®^ Q RT SuperMix for qPCR (Vazyme, Nanjing, China) according to the manufacturer’s instructions. Real-time quantitative PCR was carried out using the ChamQ SYBR qPCR Master Mix (Vazyme, Nanjing, China) on QuantStudio7 (ABI). The relative RNA expression levels were analyzed by the formula of 2^−△△Ct^. The following primers used are listed in Table 2.

**Table 2:** The primer sequences used for the qRT-PCR.

Gene	Forward	Reverse
Trio	AGGCCGAAAAGTATATGAGCAAC	GTCAAGGAGCGACTTCCCAT
E-Cadherin	TCTGCTGCTCTTGCTGTTTC	CTCTTCTCCGCCTCCTTCTT
N-Cadherin	ATCATTGCCATCCTGCTC	TCCTCCACCTTCTTCATCA
Vimentin	GAAGAGAACTTTGCCGTTG	GAAGGTGACGAGCCATTT
Snail	ACATCCGAAGCCACACG	TGGGGACAGGAGAAGGG
MMP9	ACGCAGACATCGTCATCC	CCAGGGACCACAACTCG
RUNX2	AGGCAGTTCCCAAGCATTTCATCC	TGGCAGGTAGGTGTGGTAGTGAG
COL1A1	AAAGATGGACTCAACGGTCTC	CATCGTGAGCCTTCTCTTGAG
OPN	TCACACATGGAAAGCGAGGAGTTG	ACTGTCCTTCCCACGGCTGTC
OSX	CGGCAAGAGGTTCACTCGTTCG	TGGAGCAGAGCAGGCAGGTG
OCN	CTACCTGTATCAATGGCTGGG	GGATTGAGCTCACACACCT
ALP	CTTCATAGAAGGGGAGCTGTAC	GATACAGAGTGACCGTGTCAT
GAPDH	GAAGGTGAAGGTCGGAGTC	GAGATGGTGATGGGATTTC

### Immunofluorescence assay

After transfection with siRNA control or siRNA Trio for 48 h, U2OS and 143B cells were firstly fixed with 4% PFA for 15 min at room temperature, then blocked with normal goat serum for 30 min at 37 °C and finally incubated with primary antibodies overnight at 4 °C. On the second day, the cells were washed with PBS, followed by incubation with Alexa488-conjugated secondary antibodies for 1 h at 37 °C, then washed again with PBS and subsequently stained with DAPI for 2 min. For cytoskeleton staining, the fixed cells were treated with phalloidin (Cytoskeleton) for 30 min at 37 °C. The cells were observed using the fluorescence microscope (Leica Microsystems, Ontario, Canada).

### Rho GTPase activity assay

Rac1 and RhoA activity were assessed using Rac1 Activation Assay Biochem Kit (Cytoskeleton, #BK035) and Rho Activation Assay Biochem Kit (Cytoskeleton, #BK036), respectively, following the manufacturer’s instructions. In brief, Rac1-GTP and RhoA-GTP were pulled down from total proteins using the matching beads (PAK-PBD beads for Rac1 and Rhotekin RBD beads for RhoA), then the total and active Rho GTPase was detected by Western blot with anti-Rac1 antibody (Cytoskeleton) and anti-RhoA antibody (Cytoskeleton).

### In vivo assay

5 × 10^6^ U2OS cells transfected with shCtrl or shTrio stably were inoculated subcutaneously in the flanks of BALB/C nude mice (male, 4 weeks old, *n* = 4 each group). Tumor size was measured every one week by vernier caliper through the following formula: volume (mm^3^) = *ab*^2^/2 (“*a*” is the smallest diameter, “*b*” is the largest diameter). Seven weeks after inoculation, the IVIS Spectrum Imaging System (PerkinElmer) was used to capture the GFP signal by taking photographs. Finally, mice were euthanized and tumors were harvested, weighed, photographed, and fixed in 4% PFA for future assays.

### Alkaline phosphatase (ALP) assay and Alizarin red S (ARS) staining

U2OS and 143B cells were induced for osteogenic differentiation in a normal medium supplemented with 10 mM β-glycerophosphate (Sigma, St. Louis, USA), 50 mg/ml ascorbate phosphate (Sigma), and 10 nM dexamethasone (Sigma). After 5 days of induction, cells were fixed with 4% PFA, washed with PBS, and stained using BCIP/NBT ALP Color Development Kit (Beyotime Institute of Biotechnology, Shanghai, China) following the manufacturer’s protocols. After 14 days, Alizarin red (Beyotime Institute of Biotechnology, Shanghai, China) was used to stain cells. Photos were taken using a scanner and light microscope (DMIL LED; Leica Microsystems GmbH) at ×200 magnification.

### Statistical analysis

All experiments were conducted in triplicate. Statistical analysis was analyzed using a Student’s *t*-test with GraphPad Prism 8.4.0 (GraphPad Software, La Jolla, CA, USA) and data were expressed as the mean ± SD. *p* < 0.05 and *p* < 0.01 are marked with * and **, respectively.

## Results

### Expression of Trio is elevated in OS

**Fig. 1:**
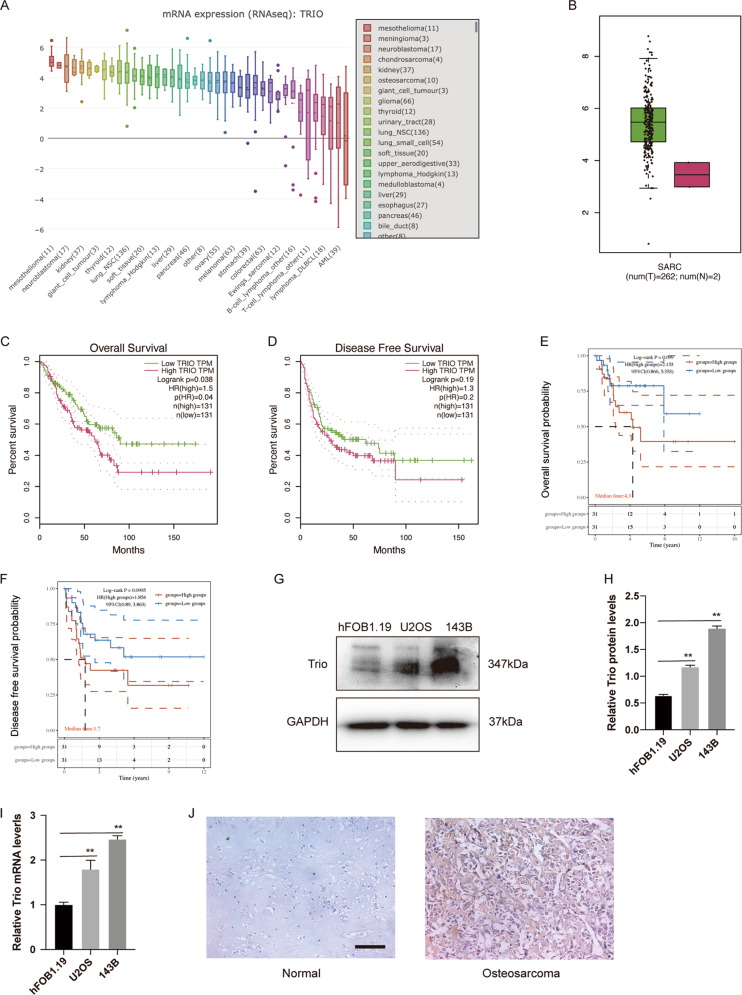
Expression of Trio is elevated in osteosarcoma. **A** The Trio mRNA levels in different cancer type cell lines from the CCLE database. **B** The expression levels of Trio in sarcoma and normal control tissues were achieved from the TCGA database. **C** and **D** Kaplan–Meier curves show the disease-free survival and the overall survival of patients with sarcoma (TCGA database) in terms of Trio gene expression. **E** and **F** Kaplan–Meier curves show the disease-free survival and the overall survival of patients with osteosarcoma (TARGET database) in terms of Trio gene expression. **G**–**I** The expression levels of Trio in hFOB1.19, U2OS,143B were measured by Western blot and qRT-PCR. **J** The expression levels of Trio in osteosarcoma and normal control tissues were detected by IHC. Representative images are shown (magnification at ×200). Data are shown as the mean ± SD. *p < 0.05, **p < 0.01. Scale bars: 100 μm.

In order to ascertain the role of Trio in OS, we firstly analyzed the data from the CCLE database and found that the mRNA level of Trio was relatively up-regulated in OS (Fig. 1A). In addition, the data from the TCGA database showed that Trio was elevated in sarcoma than normal tissues (Fig. 1B), and the prognosis of sarcoma patients with high Trio levels was worse than that with low-level ones (Fig. 1C, D). Similarly, it was suggested that Trio was associated with a poor prognosis of OS from the TARGET OS project database (Fig. 1E, F). Based on the information above, we speculated that Trio was up-regulated in OS. To test our hypothesis, we performed western blot and real-time PCR to examine the differential expression of Trio in osteoblast hFOB1.19 and OS cell lines (U2OS, 143B). As a result, Trio was significantly elevated in OS cells compared with osteoblast cells both on the transcriptional level and the protein level (Fig. 1G–I). Moreover, we also performed immunohistochemical staining to further prove that Trio was highly expressed in OS (Fig. 1J). These results suggested that Trio may play a critical role in the occurrence and development of OS.

### Trio knockdown inhibits the proliferation of OS cells

**Fig. 2:**
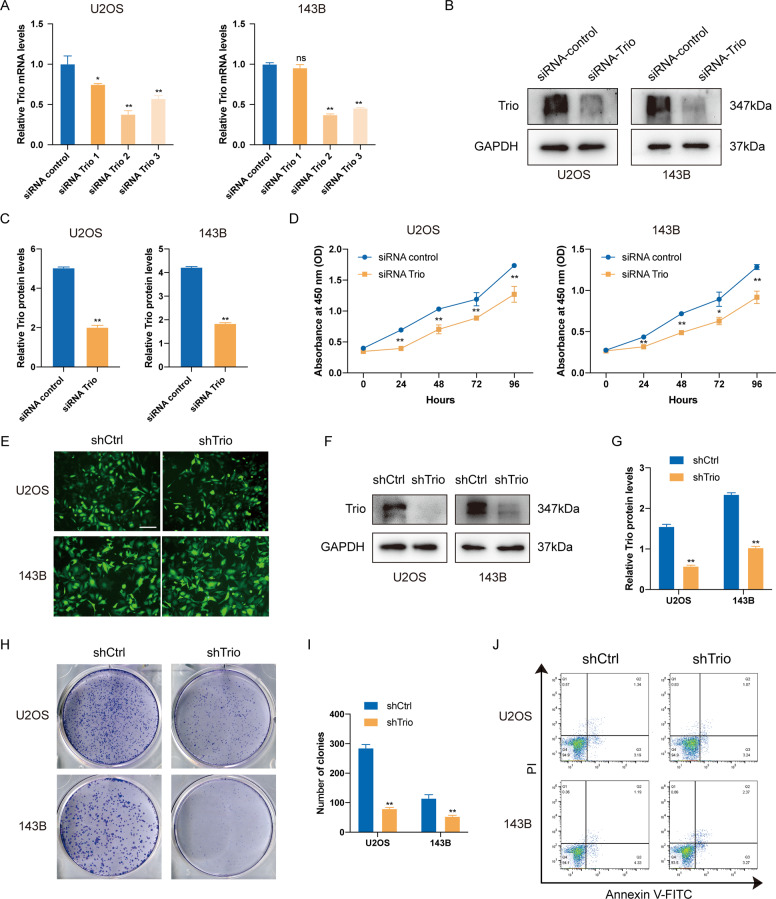
Trio knockdown inhibits the proliferation of osteosarcoma cells. **A**–**C** Efficiency of siRNA-mediated Trio knockdown in U2OS and 143B cells was measured by qRT-PCR and Western blot. **D** SiRNA-mediated Trio knockdown suppressed OS cell growth, as determined by the CCK-8 assay. **E** Efficiency of shRNA-mediated Trio knockdown in U2OS and 143B cells was detected by GFP. **F** and **G** Efficiency of shRNA-mediated Trio knockdown in U2OS and 143B cells was measured by qRT-PCR and Western blot. **H** and **I** Trio knockdown suppressed the colony formation of U2OS and 143B cells. **J** Cell apoptosis was measured by cytometry. Data are shown as the mean ± SD. *p < 0.05, **p < 0.01. Scale bars: 100 μm.

To gain further insight into the involvement of Trio in OS, we applied three siRNA targeting Trio (from GenePharma). After 48 h transfection, the silencing efficiency was detected by real-time PCR (Fig. 2A). Then the most efficient one (siRNA Trio 2) was selected and verified by western blot (Fig. 2B, C) for the subsequent assays. Firstly, we sought to examine whether Trio affected the proliferation and apoptosis of OS cells. The outcomes of CCK-8 assays demonstrated that the proliferation was impeded after Trio silencing (Fig. 2D). Meanwhile, in order to obtain a more stable and long-term knock-down level for following related experiments, U2OS and 143B cells were transfected with short hairpin RNA (shRNA) targeting Trio (shTrio-1/shTrio-2) and selected by adding puromycin (2 μg/ml) for 2 weeks. The fluorescence was observed after 72 h of transfection (Figs. 2E and S1A) and knock-down levels were detected by western blot (Figs. 2F, G, S1B), which showed that shTrio-2 could not effectively knock down Trio on the protein level. Similarly, the colony formation assays demonstrated that shTrio-1-mediated Trio deletion attenuated the proliferation of OS cells, but shTrio-2 did not significantly affect proliferation capacity (Figs. 2H, I and S2). For this reason, we selected shTrio-1 lentivirus for the following assays. There was no significant difference in apoptosis between the shCtrl group and shTrio group through flow cytometry (Fig. 2J).

### Trio knockdown inhibits OS cell migration and invasion, and affects actin cytoskeleton rearrangements

**Fig. 3:**
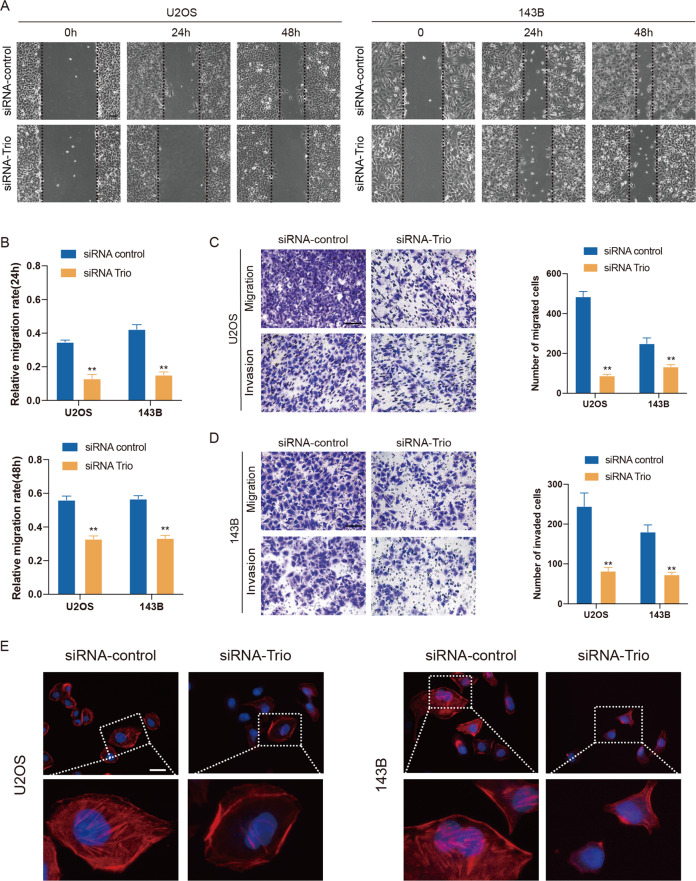
Trio knockdown inhibits osteosarcoma cell migration and invasion, and effects actin cytoskeleton rearrangements. **A** and **B** Wound-healing assay was used to measure the migration capacity of U2OS and 143B cells. Representative images are shown (magnification at ×100). **C** and **D** Transwell migration and Matrigel invasion assays were used to measure the migration and invasion ability of U2OS and 143B cells. Representative images are shown (magnification at ×200). **E** Cells of the siRNA-Trio group displayed more stress fibers and stable actin structures compared with the control group. Representative images are shown (magnification at ×400). Data are shown as the mean ± SD. *p < 0.05, **p < 0.01. Scale bars: 100 μm.

It has been established that migration and invasion are indispensable steps in the initiation of tumor metastasis. To further elucidate the role of Trio in this process, we carried out a series of experiments. Wound healing assays showed that Trio silenced cells exhibited an obvious decrease in the wound healing rate (Fig. 3A, B). In addition, transwell assays were conducted to further explore the effects of Trio on the migration and invasion potentials of U2OS and 143B cells. The results reflected that Trio silencing led to an evident decrease in both (Fig. 3C, D). Actin cytoskeleton rearrangement has been implicated in the development of tumors underlining its importance in cell motility. Accordingly, we examined whether Trio silencing altered the cytoskeleton by performing immunofluorescence assay using FITC-Phalloidin. As anticipated, the siRNA-control group had sufficient stress fibers, while the actin filament integrity was weakened in cells transfected with siRNA-Trio (Fig. 3E). Taken together, these results above suggested that Trio induced the migration and invasion potentials of OS cells.

### Trio is involved in epithelial–mesenchymal transition (EMT)

**Fig. 4:**
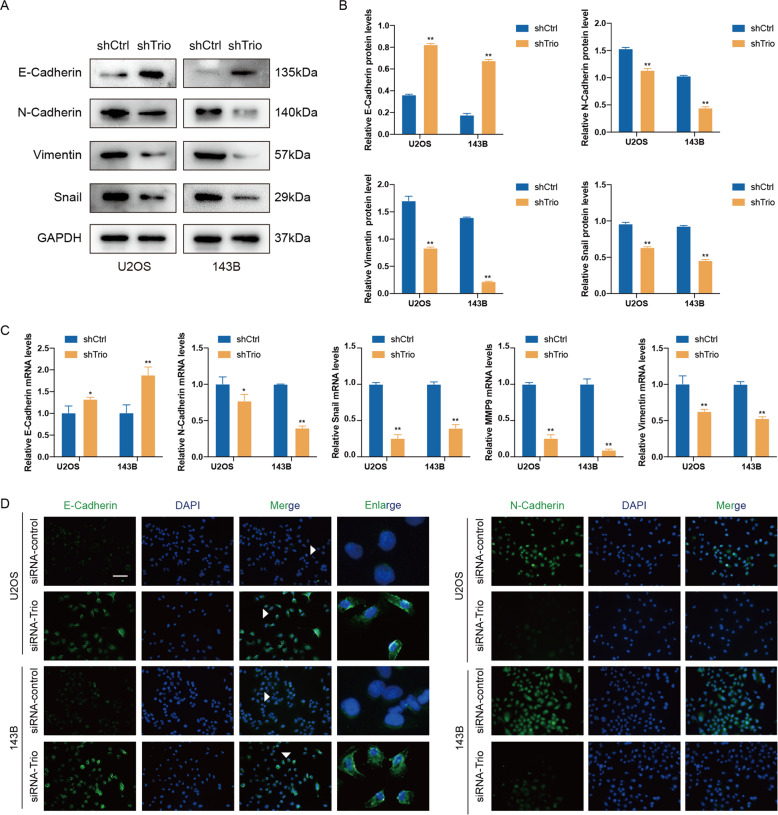
Trio knockdown suppresses EMT in osteosarcoma cells. **A** and **B** EMT markers and Snail were measured by western blotting in U2OS and 143B cells after transfection. **C** EMT markers and transcription factors (MMP9 and Snail) were measured by qRT-PCR. **D** U2OS and 143B cells were subjected to immunofluorescence with E-Cadherin and N-Cadherin antibodies. Local enlarged images showed the membrane localization of E-cad. Representative images are shown (magnification at ×200). Data are shown as the mean ± SD. *p < 0.05, **p < 0.01. Scale bars: 100 μm.

It is well known that EMT is a crucial biological process in tumor progression, in which cells transform from a polarized, closely contacted form to a form that loses polarization and becomes more motorial [33]. In view of this, it was postulated that Trio may serve as an important role in this process. As shown in Fig. 4A, C, the epithelial marker (E-cadherin) mRNA, and protein levels both exhibited an obvious increment after Trio was knocked down. Conversely, mesenchymal markers (N-cadherin, Vimentin) were down-regulated. Besides, as demonstrated in Fig. 4D, similar results were obtained by Immunofluorescence assay. In addition, to further characterize the effect of Trio on EMT, we examined the EMT-related transcription factors (MMP9, Snail), and their expression were also decreased after Trio knocked down (Fig. 4A–C). Collectively, Trio promoted OS progression in part through EMT.

### Trio knockdown promotes osteoblastic differentiation of OS cells

**Fig. 5:**
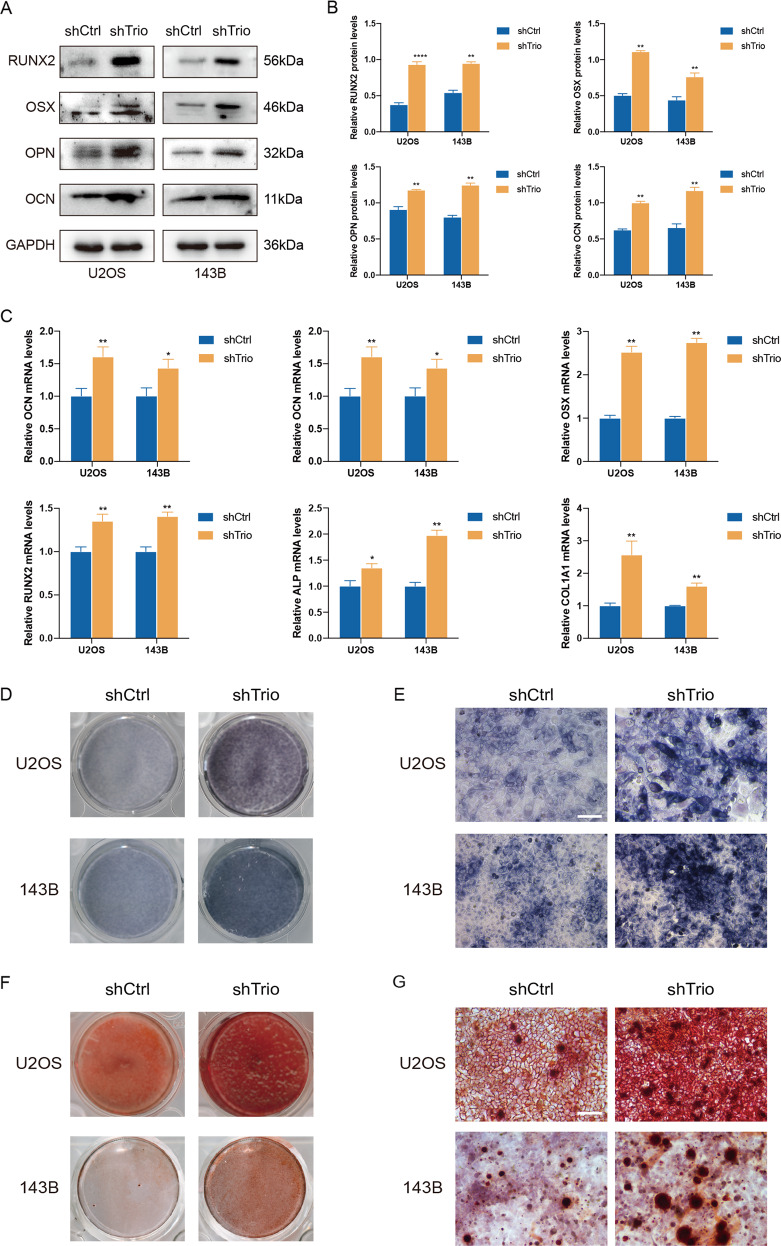
Trio knockdown promotes osteoblastic differentiation of osteosarcoma cells. **A** and **B** osteogenic markers were measured by western blotting in U2OS and 143B cells after transfection. **C** osteogenic markers and transcription factors were measured by qRT-PCR. **D**, **E** U2OS and 143B cells were stained with the ALP kit. Representative images are shown (magnification at ×200). **F**, **G** U2OS and 143B cells were stained with ARS solution. Representative images are shown (magnification at 200×). Data are shown as the mean ± SD. *p < 0.05, **p < 0.01. Scale bars: 100 μm.

Previous studies have shown that OS cells have incomplete osteoblast differentiation characteristics and loss of differentiation is a remarkable feature of osteosarcoma [34–37]. OS is associated with mutations during the differentiation of mesenchymal stem cells into osteoblasts and osteogenic differentiation of OS cells hampers OS proliferation and progression [34, 38, 39]. U2OS and 143B cells transfected with shCtrl or shTrio lentivirus stably were cultured in osteogenic media for 7 days (for ALP staining, western blot and real time PCR) or 14 days (for ARS staining). As shown in Fig. 5A–C, osteogenic differentiation markers (OPN, OCN) and osteogenic-related transcription factors (OSX, RUNX2) exhibited an upward trend both on transcriptional and protein levels after Trio knocked down. Furthermore, we performed ALP staining and ARS staining in vitro to directly observe the ALP activity and mineralization levels of cells (Fig. 5D–G). It is obvious that the osteogenic differentiation was induced though Trio deletion. In summary, Trio regulated OS progression by influencing osteogenic differentiation, which may provide us with a novel direction for the treatment of OS in the future.

### Trio knockdown suppresses tumor growth in vivo

**Fig. 6:**
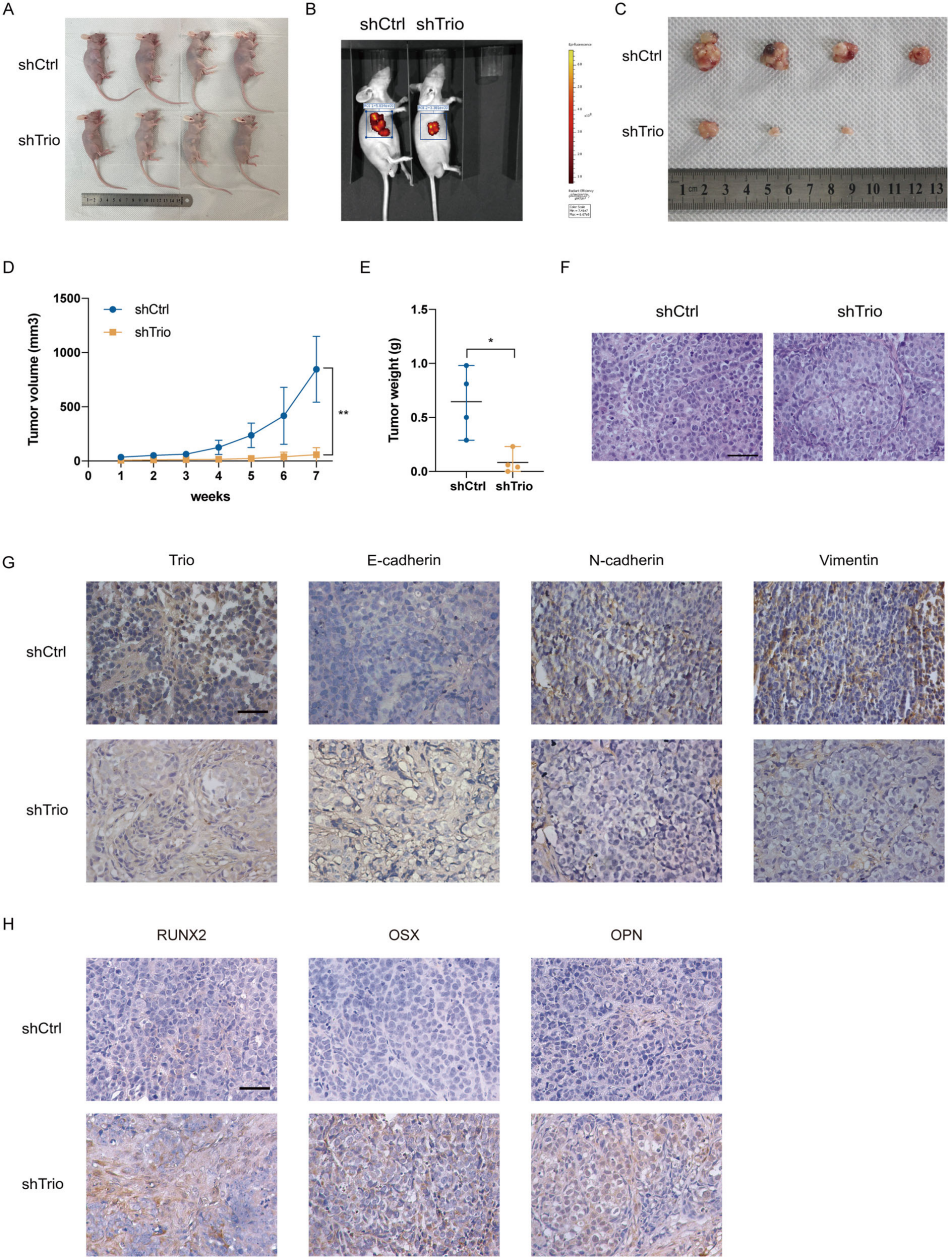
Trio knockdown suppress tumor growth in vivo. **A**, **B** After 7 weeks, the photos of BALB/C nude mice were taken. **C** Tumors were removed and photographed. **D**, **E** The volume and weight of tumor xenograft in nude mice were recorded. **F**–**H** The tumor tissues were detected by HE and IHC staining. Representative images are shown (magnification at ×400 for IHC). Data are shown as the mean ± SD. *p < 0.05, **p < 0.01. Scale bars: 50 μm.

To gain a deeper understanding of Trio’s role in OS, we built a tumor xenograft model. Four weeks-old BALB/C nude mice were chosen for experiments and injected with U2OS cells subcutaneously. During this period, the tumor volume was measured every other week (Fig. 6D). Seven weeks later, we observed that the tumor exhibited a significantly decreased growth in vivo in the shTrio group through the IVIS Spectrum Imaging System (Fig. 6A, B). Subsequently, we took the tumors out and weight them. It is evident that the tumors weighed less (Fig. 6E) and the volume increased more slowly during tumor growth in the shTrio group (Fig. 6A, C, D). Additionally, we performed IHC and H&E staining as shown in Fig. 6F–H, which were in agreement with the results in vitro. In conclusion, Trio knockdown could impede the growth of tumors and promote osteoblastic differentiation of OS cells to some extent.

### Trio regulates the progression of OS through Rac1 and RhoA

**Fig. 7:**
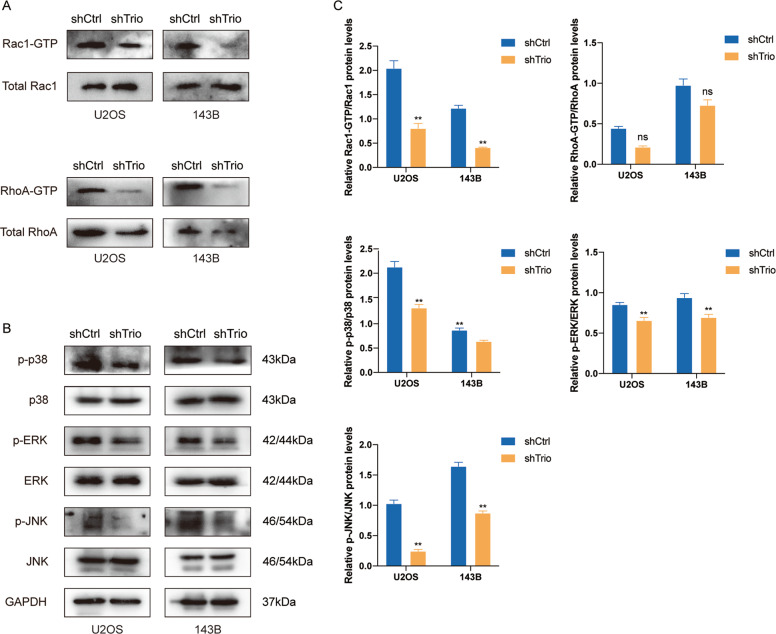
Trio regulates the progression of osteosarcoma through Rac1 and RhoA. **A** GTP-bound forms of Rac1 and RhoA were measured by pull-down assay and western blot. **B** Classical members of the MAPK pathway were analyzed by western blot. **C** Quantitative analysis of the protein levels in (**A**) and (**B**). Data are shown as the mean ± SD. *p < 0.05, **p < 0.01.

Given the established role of Trio in OS, it is necessary to further explore its mechanism of action. Since Trio contains two GEF domains, we conceived whether Trio activated RhoGTPases via GEF. Accordingly, we conducted related studies. It was observed that the expression of total Rac1 remained unchanged after Trio was knocked down, but the active Rac1 decreased significantly (Fig. 7A, C). Unexpectedly, the total RhoA was significantly reduced, so was the active RhoA (Fig. 7A, C). This discrepancy had invited speculation regarding other regulation ways that Trio acted on RhoA. We considered the possibility that Trio may indirectly regulate RhoA-GTP by controlling the total RhoA.

**Fig. 8:**
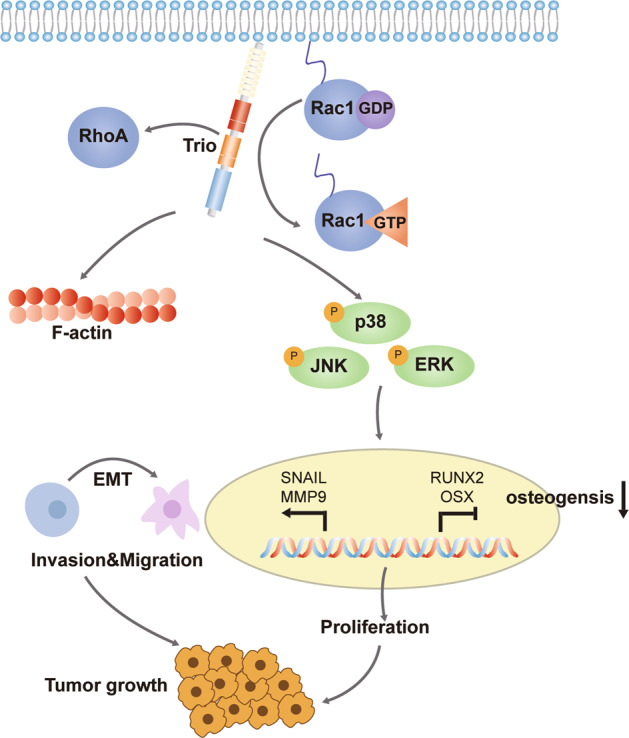
Schematic diagram of mechanism on this research. Rho-GEF Trio promotes osteosarcoma progression and inhibits osteogenic differentiation through Rac1/RhoA-mediated MAPK pathway.

There has been a number of studies indicating that the MAPK pathway was a downstream pathway of RhoGTPases and played a role in the process of osteogenic differentiation [36, 40–42], thus we pondered over whether it was also suitable for OS. Western bolt indicated that p-p38, p-JNK, p-ERK were all down-regulated in the shTrio group (Fig. 7B, C). In general, all results above revealed that Trio could regulate the MAPK pathway via Rac1 and RhoA to participate in EMT and osteogenic differentiation, eventually influencing the proliferation, migration, invasion, and growth of OS (Fig. 8).

## Discussion

Herein, we proved that Trio could be involved in promoting the progression of OS both in vitro and vivo for the first time. In addition, since OS cells originate from osteoblasts and several studies have pointed out that differentiation therapy may be a novel promising approach intended to restore normal programs of differentiation [35, 43]. We examined whether Trio affected the osteogenic differentiation of OS cells. As a result, we revealed that Trio could inhibit OS’s osteogenic differentiation both in vitro and vivo, supporting the notion above. However, it remains to be elucidated in more detail about the exact mechanism in the future.

A number of studies have put forward that Trio is instrumental in a variety of tumors [15, 19–21], however, there are few types of research on its regulatory mechanism. In this study, we outlined a novel signaling pathway responsible for the effects of Trio on OS. Besides Trio, other RhoGEFs also have been reported to have an important function in cancer [44–46]. Accordingly, we considered the possibility that most RhoGEFs may play crucial roles in oncogenesis, which provided a new direction for further research. Moreover, Trio was thought to activate Rac1 and RhoA through distinct ways in our study. The discrepancies may be explained in part by the fact that Rac1 and RhoA display opposite behaviors and spatial localization in cell migration [44, 47]. Therefore, we postulated that there may be a balance between Rac1 and RhoA.

It is well known that the MAPK pathway plays a crucial role in osteogenic differentiation and tumor progression [48–50]. Furthermore, several studies have proved that the MAPK pathway is a downstream pathway of RhoGTPase [36, 40], but there are relatively few studies in OS to date. Thus, we explored whether this regulatory axis exists in OS. Eventually, we demonstrated that the Rac1/RhoA-p38/ERK/JNK axis did play a role in OS.

EMT is a typical biological process in tumor development [51]. In this process, cells will lose their cell polarity, cell–cell contact, and adhesion, then gain migratory and invasive abilities [52–54]. Studies have shown that RhoGTPase involves in EMT [55–59], in which RhoA promotes the formation of stress fibers, whereas Rac1 and Cdc42 promote the lamellipodia and filopodia formation [53, 60]. For this reason, there was a hypothesis that Trio regulated EMT through Rac1/RhoA-p38/ERK/JNK pathway. To test this hypothesis, we performed some assays to confirm it.

In conclusion, we have found the Trio-Rac1/RhoA-p38/ERK/JNK regulatory axis which induced EMT and impeded osteogenic differentiation in OS cells, leading to the progression of OS and bone destruction eventually. This regulatory axis may serve as a therapeutic target in the prevention and treatment of OS in the future.

## Supplementary information

Authorship corrections

Supplementary figure

Reproducibility Checklist

## Data Availability

The data that support the findings of this study are available on request from the corresponding author.
