# Supplementary material for: Rho-GEF Trio regulates osteosarcoma progression and osteogenic differentiation through Rac1 and RhoA
Source: Cell Death Dis. 2021 Dec 11;12(12):1148. doi: 10.1038/s41419-021-04448-3 (PMC8664940; doi:10.1038/s41419-021-04448-3)
Supplement: Supplementary file 1 — Authorship corrections [file 41419_2021_4448_MOESM1_ESM.pdf]

Re: Authorship corrections for CDDIS-21-2895

共2封

我 我 09:49 已发送
发给 wangjunyisoso@163.com

Dear Junyi Wang：

In view of Yuhuan Ma's contribution in revision for manuscript CDDIS-21-2895 entitled "Rho-GEF Trio regulates osteosarcoma progression and osteogenic differentiation through Rac1 and RhoA" , I intended to add him as a co-author and hope to obtain your consent.

Sincerely,

Junqing Ma

再次编辑 •••

王 王君仪 09:54
发给 我

I agree to add Yuhuan Ma as a co-author for the manuscript CDDIS-21-2895 entitled "Rho-GEF Trio regulates osteosarcoma progression and osteogenic differentiation through Rac1 and RhoA".

Re: Authorship corrections for CDDIS-21-2895

共2封

我 我 10:10 已发送
发给 YANA袁

Dear Lichan Yuan：

In view of Yuhuan Ma's contribution in revision for manuscript CDDIS-21-2895 entitled "Rho-GEF Trio regulates osteosarcoma progression and osteogenic differentiation through Rac1 and RhoA" , I intended to add him as a co-author and hope to obtain your consent.

Sincerely,

Junqing Ma

再次编辑 •••

YANA袁 10:12
发给 我

I agree to add Yuhuan Ma as a co-author for the manuscript CDDIS-21-2895 entitled "Rho-GEF Trio regulates osteosarcoma progression and osteogenic differentiation through Rac1 and RhoA".

Re:Authorship corrections for CDDIS-21-2895

共2封

我 我 09:46 已发送
发给 xuxiaohong1207@126.com

Dear Xiaohong Xu:

In view of Yuhuan Ma's contribution in revision for manuscript CDDIS-21-2895 entitled "Rho-GEF Trio regulates osteosarcoma progression and osteogenic differentiation through Rac1 and RhoA" , I intended to add him as a co-author and hope to obtain your consent.

Sincerely,

Junqing Ma

再次编辑 •••

徐 徐小红 10:20
发给 我

I agree to add Yuhuan Ma as a co-author for the manuscript CDDIS-21-2895 entitled "Rho-GEF Trio regulates osteosarcoma progression and osteogenic differentiation through Rac1 and RhoA".

Re: Authorship corrections for CDDIS-21-2895

共2封

我 我 09:49 已发送
发给 wangjunyisoso@163.com

Dear Junyi Wang：

In view of Yuhuan Ma's contribution in revision for manuscript CDDIS-21-2895 entitled "Rho-GEF Trio regulates osteosarcoma progression and osteogenic differentiation through Rac1 and RhoA" , I intended to add him as a co-author and hope to obtain your consent.

Sincerely,

Junqing Ma

再次编辑 •••

王 王君仪 09:54
发给 我

I agree to add Yuhuan Ma as a co-author for the manuscript CDDIS-21-2895 entitled "Rho-GEF Trio regulates osteosarcoma progression and osteogenic differentiation through Rac1 and RhoA".

CDDIS-21-2895

共2封

我 我 10:07 已发送
发给 hongleilei39@163.com

Dear Leilei Hong：

In view of Yuhuan Ma's contribution in revision for manuscript CDDIS-21-2895 entitled "Rho-GEF Trio regulates osteosarcoma progression and osteogenic differentiation through Rac1 and RhoA" , I intended to add him as a co-author and hope to obtain your consent.

Sincerely,

Junqing Ma

再次编辑 •••

H hongleilei39 10:47
发给 我

I agree to add Yuhuan Ma as a co-author for the manuscript CDDIS-21-2895 entitled "Rho-GEF Trio regulates osteosarcoma progression and osteogenic differentiation through Rac1 and RhoA".
