# Supplementary material for: Rho-GEF Trio regulates osteosarcoma progression and osteogenic differentiation through Rac1 and RhoA
Source: Cell Death Dis. 2021 Dec 11;12(12):1148. doi: 10.1038/s41419-021-04448-3 (PMC8664940; doi:10.1038/s41419-021-04448-3)
Supplement: Supplementary file 2 — Supplementary figure [file 41419_2021_4448_MOESM2_ESM.docx]

**Rho-GEF Trio regulates osteosarcoma progression and osteogenic differentiation through Rac1 and RhoA**

Junyi Wang^1*^, Lichan Yuan^1*^, Xiaohong Xu^2^, Zhongyin Zhang^1^, Leilei Hong^1^, Junqing Ma^1^

^1^Jiangsu Key Laboratory of Oral Diseases, Nanjing Medical University, 140 Hanzhong Road, Nanjing 210029, China.

^2^MYOUR Dental, 1661 Changning Road, Shanghai 200050, China.

Correspondence: Junqing Ma, Nanjing Medical University, 140 Hanzhong Road, Nanjing 210029, China. E-mail: jma@njmu.edu.cn.

^*^These authors contributed equally to this work.

**Supplemental Figures**

**Figure S1**


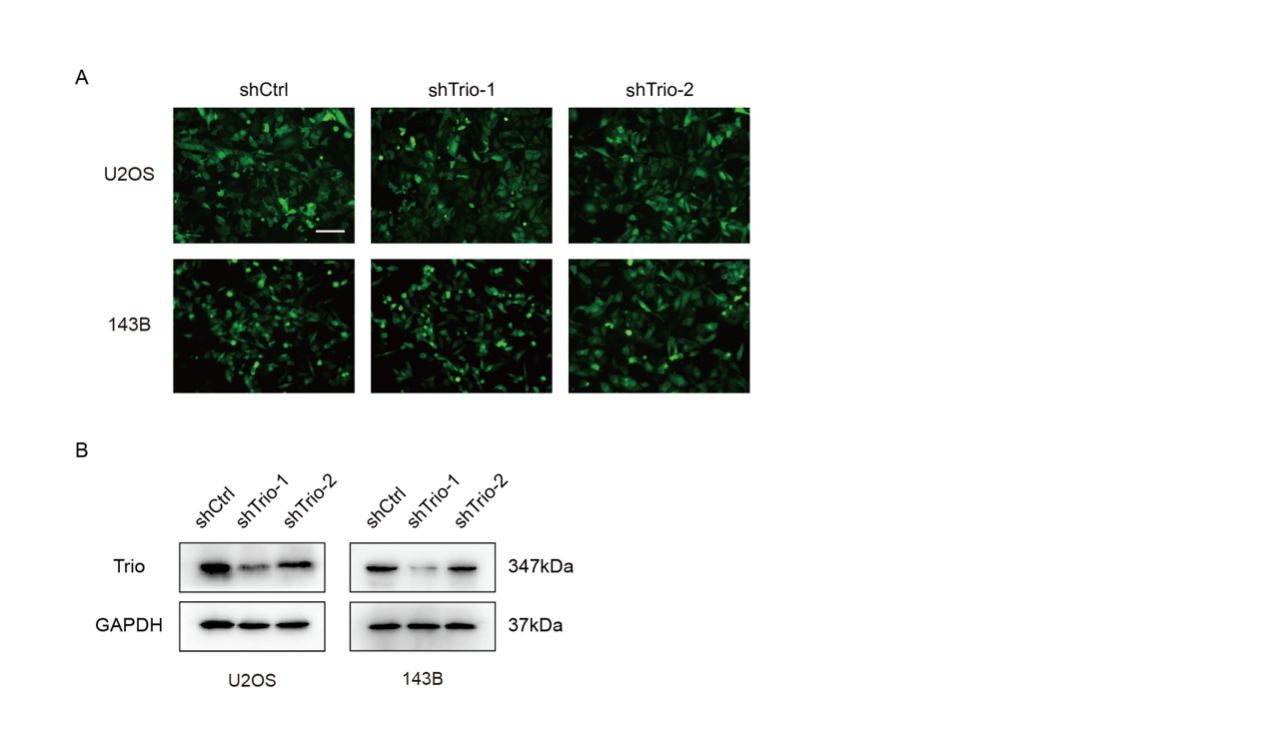


Fig.S1 Expression of Trio after 72h transfection with shCtrl or shTrio lentiviruses. (A) GFP was observed after 72h transfection. (B) Efficiency of shRNA-mediated Trio knockdown was measured by Western blot.

**Figure S2**


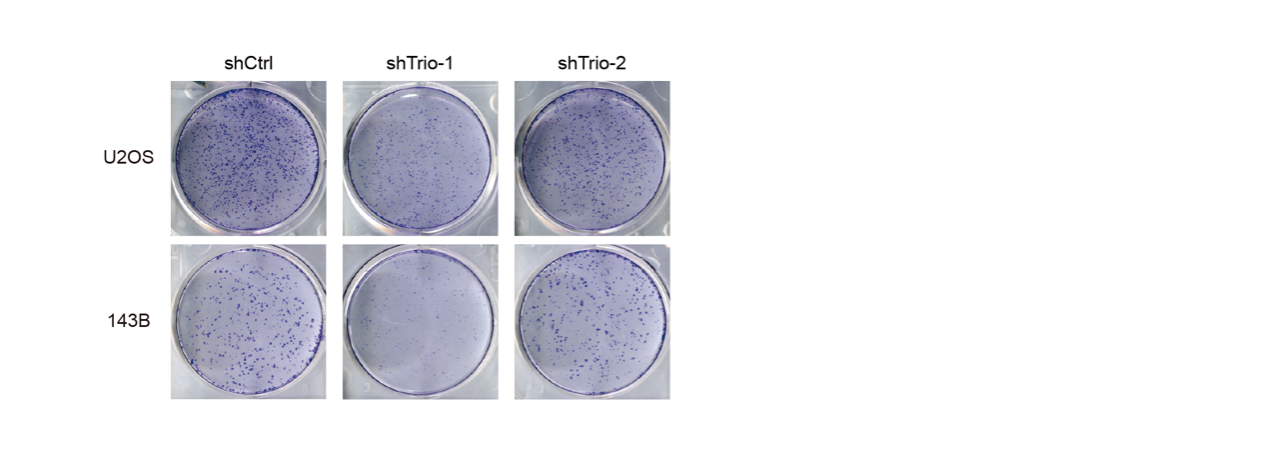


Fig.S2 The effect of shRNA-mediated Trio knockdown on colony formation.
